# Supplementary material for: Fungal Glycoside Hydrolases Display Unique Specificities for Polysaccharides and Staphylococcus aureus Biofilms
Source: Microorganisms. 2023 Jan 23;11(2):293. doi: 10.3390/microorganisms11020293 (PMC9964650; doi:10.3390/microorganisms11020293)
Supplement: Supplementary file 1 [file microorganisms-11-00293-s001.zip › microorganisms-2162068-supplementary.pdf]

Supplemental Data

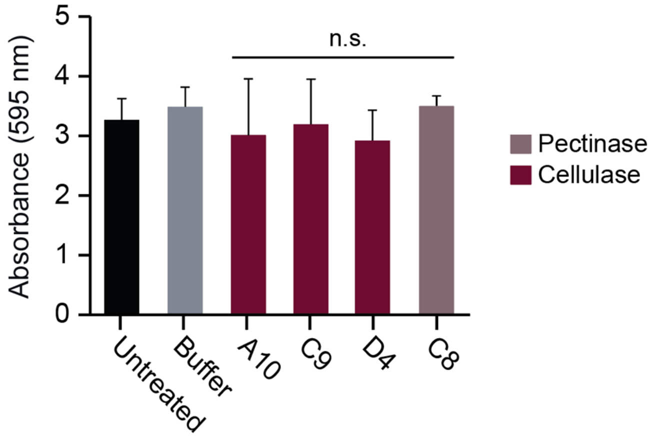

Figure S1. GHs identified by polysaccharide agar screens do not disrupt biofilms. *S. aureus* biofilms were grown on polystyrene treated with spent growth media from four strains of GH-expressing *P. pastoris*. All wells were stained with 0.1% crystal violet, and the absorbance was measured at OD_{595} (n=2).

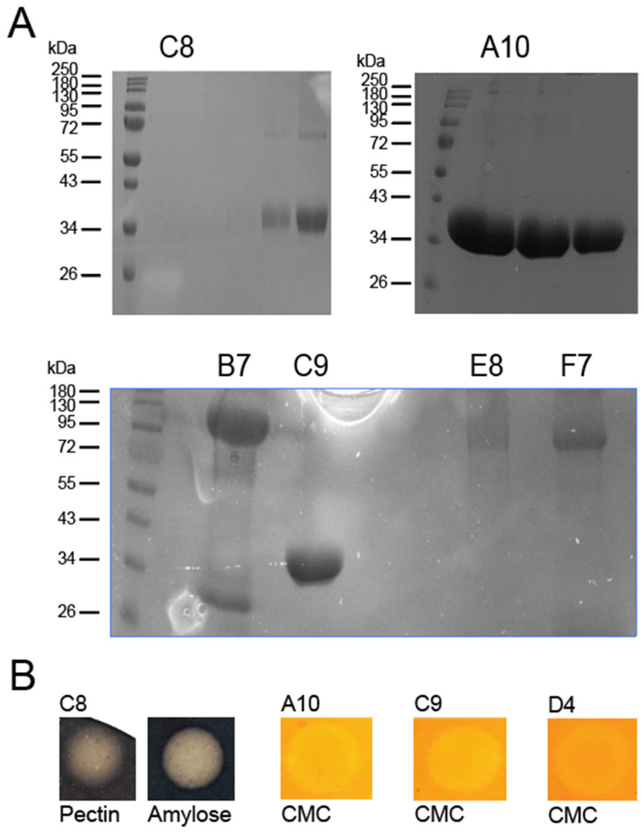

Figure S2. The purification and activity of recombinant GHs expressed by *P. pastoris*. (A) SDS-PAGE gels loaded with fractions containing purified GHs E8 (α-xylosidase, Acc number), C8 (pectinase, AN3390.2), A10 (cellulase, AN1285.2), C9 (cellulase, AN3418.2), D4 (cellulase, AN5214.2), F7 (β-xylosidase, AN8401.2), and B7 (β-xylosidase, AN8401.2). (B) After incubation with GH, agar was stained with either Congo Red or Lugol's solution to detect hydrolysis. Concentrations are direct elution fractions all >1 mg mL^{-1}.

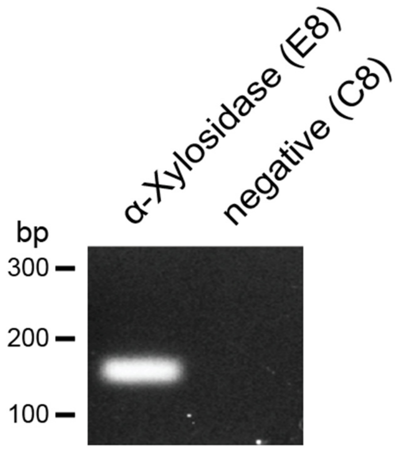

Figure S3. Confirmation of E8 α-xylosidase gene by PCR. Agarose gel electrophoresis of PCR products targeting the α-xylosidase gene (accession no. AN7505.2). Genomic DNA from *P. pastoris* from well E8 (Lane 1) and C8 as a negative control (Lane 2). The expected product from amplifying the α-xylosidase gene is 173 bp.

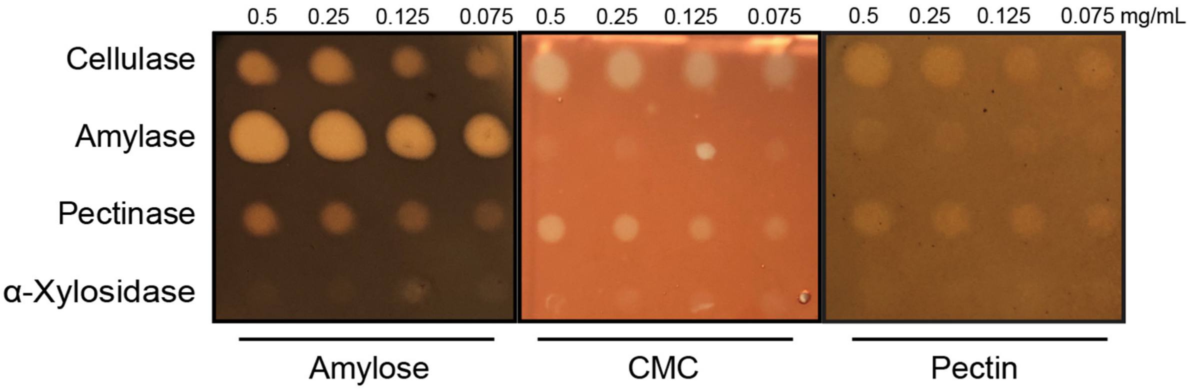

Figure S4. Purified α-xylosidase is not active in the hydrolysis of amylose, CMC, or pectin. Commercial preparations of different GHs and purified recombinant E8 α-xylosidase (A), and *A. thermomutatus* α-xylosidase (B), were pinned at different concentrations on 1% w/v amylose, CMC, and pectin containing agar. Plates were stained with Congo Red and Lugol's solution to detect hydrolysis.

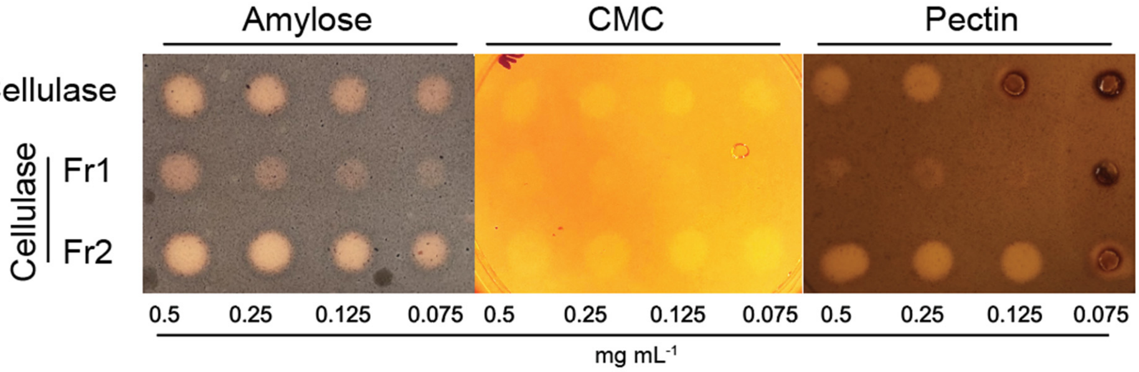

Figure S5. Commercial preparations of cellulase and amylase contain contaminating proteins with different GH activities. Commercial cellulase, and fraction 1 (glucoamylase; UniProt accession number A0A117E3H6), and

fraction 2 (β-xylanase; UniProt accession number A0A100I6F6) were spotted at different concentrations on amylose, CMC, and pectin-containing agar before staining with either Congo Red and or Lugol's solution.

Table S1. Recombinant GHs created by Bauer et al. used in this study.

FGSC #	Genbank Accession #	Well
9925	AN1542.2	A1
10060	AN0393.2	A2
10061	AN0452.2	A3
10062	AN0494.2	A4
10063	AN712.2	AS
10064	AN741.2	A6
10065	AN787.2	A7
10066	AN941.2	AS
10067	AN1277.2	A9
10068	ANl285.2	AI0
10069	AN155J.2	A11
10070	AN1571.2	A12
10071	AN1602.2	Bl
10072	AN1804.2	B2
10073	AN1818.2	B3
10074	AN2206.2	B4
10075	AN2227.2	B5
10076	AN2331.2	B6
10077	AN2359.2	B7
10078	AN2385.2	B8
10079	AN2528.2	B9
10080	AN2559.2	B10
10081	AN2612.2	B11
10082	AN3044.2	B12
10083	AN3049.2	C1
10084	AN3201.2	C2
10085	AN3294.2	C3
10086	AN3297.2	C4
10087	AN3337.2	C5
10088	AN3358.2	C6
10089	AN3368.2	C7
10090	AN3390.2	C8
10091	AN3418.2	C9
10092	AN3613.2	C10

10093	AN3777.2	C11
10094	AN4372.2	C12
10095	AN4700.2	D1
10096	AN4843.2	D2
10097	AN5176.2	D3
10098	AN5214.2	D4
10099	AN5267.2	D5
10100	AN5282.2	D6
10101	AN5361.2	D7
10102	AN5727.2	D8
10103	AN6093.2	D9
10104	AN6352.2	D10
10105	AN6395.2	D11
10106	AN6427.2	D12
10107	AN6470.2	E1
10108	AN7135.2	E2
10109	AN7152.2	E3
10110	AN7180.2	E4
10111	AN7345.2	E5
10112	AN7349.2	E6
10113	AN7413.2	E7
10114	AN7505.2	E8
10115	AN7533.2	E9
10116	AN7541.2	E10
10117	AN7624.2	E11
10118	AN7646.2	E12
10119	AN7908.2	F1
10120	AN7950.2	F2
10121	AN8007.2	F3
10122	AN8138.2	F4
10123	AN8149.2	F5
10124	AN8327.2	F6
10125	AN8401.2	F7
10126	AN8453.2	F8
10127	AN8761.2	F9
10128	AN9035.2	F10
10129	AN9045.2	F11
10130	AN9134.2	F12
10131	AN9286.2	G1

10132	Afu8g06890	G2
10133	NCU09102.7	G3
10134	AN3556.2	G4

Table S2. Primers used in this study. Underlines sequence represents regions of sequence homology used during the construction of plasmids by recombineering.

Primer	Sequence	Notes
JEx0015	GCTGAATTCACGTGGCCCAGCCGGCCGTCT<br>ATGAAGTTTACCGAGGGAATGTGG	Forward primer used to amplify the fragment containing the *A. thermomutatus* α-xylosidase gene.
JEx002	GGCGGCCGCCGCGGCTCGAGGTACCGATCC<br>TCAATGATGATGATGATGATGGTCGACG	Reverse primer used to amplify the fragment containing the *A. thermomutatus* α-xylosidase gene.
JEx003	GGATCGGTACCTCGAGCCG	Forward primer used to amplify a region of the pPICZα A vector.
JEx004	AAACTGTCAGTTTTGGGCCATTTGGGGAAC	Reverse primer used to amplify a region of the pPICZα A vector.
JEx005	GTTCCCCAAATGGCCCAAAACTGACAGTTT<br>GATGCGGTATTTTCTCCTTACGC	Forward primer used to amplify the fragment containing a URA3 gene and yeast 2-um origin.
JEx006	CGAAAAGTGCCACCTGAACG	Reverse primer used to amplify the fragment containing a URA3 gene and yeast 2-um origin.
JEx007	ACAGATGCTTCGTTCAGGTGGCACTTTTCG<br>AAACGCTGTCTTGGAACCTAATATGACAAAAGC	Forward primer used to amplify a region of the pPICZα A vector.
JEx0016	AGACGGCCGGCTGGGC	Reverse primer used to amplify a region of the pPICZα A vector.
JEx009	CTAATATGACAAAAGCGTGATCTCATCC	Forward primer used to amplify a region from the recombineering constructed plasmid, which is then used to transform *P. pastoris.*
JEx010	CTATTTGACCCCACACTCAGAAAGC	Reverse primer used to amplify a region from the recombineering constructed plasmid which is then used to transform *P. pastoris.*
JEx011	ATTTAGAAGGGGATTTCGATGTTGC	Forward primer used to amplify the *A. thermomutatus* α-xylosidase gene after it has been genomically integrated into *P. pastoris*.
JEx012	CCCCTACCACAAGATATTCATCAGC	Reverse primer used to amplify the *A. thermomutatus* α-xylosidase gene after it has been genomically integrated into *P. pastoris*.
JExREV01	CGCGACTTATCTGTAGTTTGG	Reverse sequencing primer for the *A. thermomutatus* α-xylosidase gene integrated into *P. pastoris*.
JExREV02	TAAGACCATGAAGGCACAAG	Reverse sequencing primer for the *A. thermomutatus* α-xylosidase gene integrated into *P. pastoris*.
JExREV03	AAGTGTGCTACGAACCAAGC	Reverse sequencing primer for the *A. thermomutatus* α-xylosidase gene integrated into *P. pastoris*.
JExREV04	GTGGGAGCTCAGTAATCCAA	Reverse sequencing primer for the *A. thermomutatus* α-xylosidase gene integrated into *P. pastoris*.
JExREV05	ATCGGCAAAGTATCAAAGCC	Reverse sequencing primer for the *A. thermomutatus* α-xylosidase gene integrated into *P. pastoris*.

JExFOR02	GTTGTCTATGGGAAAACACCA	Forward sequencing primer for the *A. thermomutatus* α-xylosidase gene integrated into *P. pastoris*.
JExFOR03	ATGGTTCTGTGTGGCAGTG	Forward sequencing primer for the *A. thermomutatus* α-xylosidase gene integrated into *P. pastoris*.
JExFOR04	GTTACATATTTTGGGCCTCG	Forward sequencing primer for the *A. thermomutatus* α-xylosidase gene integrated into *P. pastoris*.
JExFOR05	GAAATGGACGTTGGAGGTC	Forward sequencing primer for the *A. thermomutatus* α-xylosidase gene integrated into *P. pastoris*.

Table S3. Purified α-xylosidase retains activity. Purified enzyme was placed in buffer containing a final concentration of 0.1% w/v 4-Nitrophenyl α-D-xylopyranoside. Presence of released o-nitrophenol by measurement of 405 nm absorbance was taken 50 seconds after reaction initiation. Reactions took place at room temperature.

**α-xylosidase (mg mL^{-1})**	**0.25**	**0.125**	**0.05**	**0.005**
***A. nidulans* (E8) (405nm abs)**	0.123	0.078	0.044	0
***A. thermomutatus* (405nm abs)**	0.022	0.002	0	0

Table S4. Potential proteins identified through mass spectrometry for cellulase fraction 1. Protein threshold set at 1.0% FDR, and individual peptide threshold set at 95% probability. (Accession refers to UniProt accession number, %Spec refers to protein percentage of total spectra, #Pep refers to the exclusive unique peptide count, #Unique refers to the exclusive unique spectrum count, #Spec refers to the exclusive spectrum count, %Cov refers to the percentage of amino acids identified. *Most likely enzyme ID based on size, coverage and spectral data.

Protein annotation	Accession	%Spec	#Pep	#Unique	#Spec	%Cov	m.w.
Glucanase	A0A117DZQ3	2.29%	16	32	604	44.25%	48296 Da
Glucoamylase*	A0A117E3H6	0.23%	13	20	60	27.06%	68728 Da
Peptide hydrolase	A0A117DVZ2	0.14%	11	15	38	19.21%	68831 Da
Endo-β-1,4-mannanase F	A0A100ILF3	0.12%	6	12	32	22.10%	56129 Da
Exo-β-1,3-glucanase	A0A117DW84	0.11%	6	13	30	11.96%	99332 Da
GPI-anchored cell wall organization protein Ecm33	A0A117DX26	0.11%	6	9	28	18.20%	41158 Da
β-xylanase	A0A100I6F6	0.09%	10	14	25	29.91%	35060 Da
α-1,3-glucanase/mutanase	A0A100ICW4	0.09%	10	14	24	17.06%	54579 Da
α-galactosidase	A0A100I971	0.09%	8	13	24	21.08%	49094 Da
Probable endo-β-1,4-glucanase	A0A117DY27	0.08%	6	11	22	10.54%	36715 Da
Glucanase	A0A100IHS6	0.07%	6	10	19	25.50%	42201 Da
Alpha-L-arabinofuranosidase	A0A117DZC8	0.06%	6	8	15	18.84%	52598 Da
Xylosidase: arabinofuranosidase	A0A100IHT3	0.05%	8	8	14	13.81%	60310 Da

Fungal specific transcription factor	A0A100IN41	0.05%	7	8	14	6.31%	128083 Da
1,3-β-glucanosyltransferase	A0A100IR34	0.05%	5	6	14	12.59%	56966 Da
1,3-β-glucanosyltransferase	A0A100IIK3	0.05%	6	8	14	20.16%	52125 Da
α-1,2-Mannosidase	A0A117DW82	0.05%	7	7	13	15.56%	63073 Da
1,3-β-glucanosyltransferase	A0A100INU2	0.05%	6	6	13	5.99%	114558 Da
α-amylase	A0A124BXE9	0.05%	5	7	13	8.13%	127175 Da

Table S5. Potential proteins identified through mass spectrometry for cellulase fraction 2. Protein threshold set at 1.0% FDR, and individual peptide threshold set at 95% probability. (Accession refers to UniProt accession number, %Spec refers to protein percentage of total spectra, #Pep refers to the exclusive unique peptide count, #Unique refers to the exclusive unique spectrum count, #Spec refers to the exclusive spectrum count, %Cov refers to the percentage of amino acids identified. *Most likely enzyme ID based on size, coverage and spectral data.

Protein annotation	Accession	%Spec	#Pep	#Unique	#Spec	%Cov	m.w.
Endo-β-1,4-mannanase F	A0A100ILF3	1.02%	11	25	266	30.34%	56129 Da
β-xylanase*	A0A100I6F6	0.24%	15	23	63	53.58%	35060 Da
Probable glucan endo-1,3-β-glucosidase eglC	A0A117E1K1	0.18%	6	6	48	11.92%	45945 Da
Probable endo-β-1,4-glucanase B	A0A117DY27	0.14%	9	16	37	24.70%	36715 Da
α-L-arabinofuranosidase	A0A100I6G0	0.14%	6	13	36	26.81%	36039 Da
Glucanase	A0A117DZQ3	0.13%	7	10	35	22.35%	48296 Da
Endo-β-1,4-glucanase D	A0A100IID9	0.10%	7	9	27	12.75%	41004 Da
Endo-β-1,4-glucanase D	A0A117E071	0.10%	7	14	27	32.75%	36762 Da
WD repeat protein	A0A124BWD4	0.07%	6	8	19	4.66%	150204 Da
Feruloyl esterase A	W6GEY2	0.07%	5	9	18	21.35%	30374 Da
Pectinesterase	A0A100IC32	0.07%	6	8	17	26.91%	34680 Da
α-1,3-glucanase/mutanase	A0A100ICW4	0.05%	6	8	14	15.67%	54579 Da
Endopolygalacturonases	A0A100I4L3	0.05%	6	7	13	18.78%	37955 Da
Endoglucanase	A0A100IKG6	0.04%	5	6	11	18.41%	25765 Da
